# High-frequency brain networks undergo modular breakdown during epileptic seizures

**DOI:** 10.1111/epi.13413

**Published:** 2016-05-25

**Authors:** Stefan Fuertinger, Kristina Simonyan, Michael R. Sperling, Ashwini D. Sharan, Farid Hamzei-Sichani

**Affiliations:** * Department of Neurology, Icahn School of Medicine at Mount Sinai, New York, New York, U.S.A.; † Department of Otolaryngology, Icahn School of Medicine at Mount Sinai, New York, New York, U.S.A.; ‡ Department of Neurology, Sidney Kimmel College of Medicine, Thomas Jefferson University, Philadelphia, Pennsylvania, U.S.A.; § Department of Neurosurgery, Sidney Kimmel College of Medicine, Thomas Jefferson University, Philadelphia, Pennsylvania, U.S.A.; ¶ Department of Neurosurgery, Icahn School of Medicine at Mount Sinai, New York, New York, U.S.A.

**Keywords:** High-frequency oscillations, Epileptogenesis, Functional connectome, Cortical network

## Abstract

**Objective:**

Cortical high-frequency oscillations (HFOs; 100–500 Hz) play a critical role in the pathogenesis of epilepsy; however, whether they represent a true epileptogenic process remains largely unknown. HFOs have been recorded in the human cortex but their network dynamics during the transitional period from interictal to ictal phase remain largely unknown. We sought to determine the high-frequency network dynamics of these oscillations in patients with epilepsy who were undergoing intracranial electroencephalographic recording for seizure localization.

**Methods:**

We applied a graph theoretical analysis framework to high-resolution intracranial electroencephalographic recordings of 24 interictal and 24 seizure periods to identify the spatiotemporal evolution of community structure of high-frequency cortical networks at rest and during multiple seizure episodes in patients with intractable epilepsy.

**Results:**

Cortical networks at all examined frequencies showed temporally stable community architecture in all 24 interictal periods. During seizure periods, high-frequency networks showed a significant breakdown of their community structure, which was characterized by the emergence of numerous small nodal communities, not limited to seizure foci and encompassing the entire recorded network. Such network disorganization was observed on average 225 s before the electrographic seizure onset and extended on average 190 s after termination of the seizure. Gamma networks were characterized by stable community dynamics during resting and seizure periods.

**Significance:**

Our findings suggest that the modular breakdown of high-frequency cortical networks represents a distinct functional pathology that underlies epileptogenesis and corresponds to a cortical state of highest propensity to generate seizures.

Cortical high-frequency oscillations (HFOs; 100–500 Hz) have an important physiologic function in cognition and have been a subject of intense basic and clinical research in recent years.^1–3^ Emergence of HFOs prior to ictal discharges was documented in several in vivo and in vitro studies.^4,5^ In contrast to other epileptiform discharges, such as sharp waves or spikes, HFOs are often a reliable marker of the epileptogenic area,^6,7^ as resection of HFO-generating areas has been shown to correlate with higher rates of seizure freedom than resection of the seizure-onset zone alone.^6^ However, despite their clinical importance, the basic network dynamics of these oscillations remain only partially understood. Specifically, it is unclear how focal HFOs generated in small volumes of human cortical tissue on the scale of a cortical column^2,8,9^ can synchronize across large areas of the cortex. The paucity of this knowledge, in turn, hinders the development of novel approaches for monitoring the seizure onset, localization of the core epileptogenic network, and advancements of pharmacologic and/or surgical treatments.

In this study, we sought to identify dynamic transitions in the topologic structure of the cortical functional connectome at different high-frequency bands using intracranial electroencephalography (iEEG) recordings in patients with intractable epilepsy. We defined our data according to different pathophysiologic states, including resting (i.e., interictal) and seizure (i.e., preictal, ictal, and postictal) states. We used graph theoretical analysis of cortical activity recorded by intracranial electrodes to fully characterize network-wide changes of the functional structure underlying seizure generation. Based on prior studies suggesting likely distinct cellular and network mechanisms underlying the generation of high-frequency and gamma oscillations during epileptogenesis,^10^ we examined the spatiotemporal dynamics of cortical brain networks at gamma (30–80 Hz) and high-frequency bands, including ripple (80–250 Hz), fast-ripple (250–500 Hz), and HFO (100–500 Hz) frequency ranges. We hypothesized that the presence of HFOs in the epileptogenic regions would induce measurable changes in the architecture of functional brain networks that precede the onset of seizures.

## Methods

### Subjects

To estimate the number of resting and seizure periods, we reviewed a recent study on the spatial localization of network connectivity within the seizure-onset zone (SOZ) relative to areas outside the SOZ.^11^ Using these values, two-sample *t*-test calculations indicated that a sample size of a total of 24 seizure and 24 resting periods would provide 80% power to detect network differences at a 0.01 significance level. We selected 24 seizure episodes and corresponding 24 interictal periods in four adult male patients with medically intractable epilepsy, whose implanted subdural grids were of similar size and location to ensure topologic consistency of recordings and subsequent statistics across patients (Table S1 and Fig. S1).

**Figure 1.**
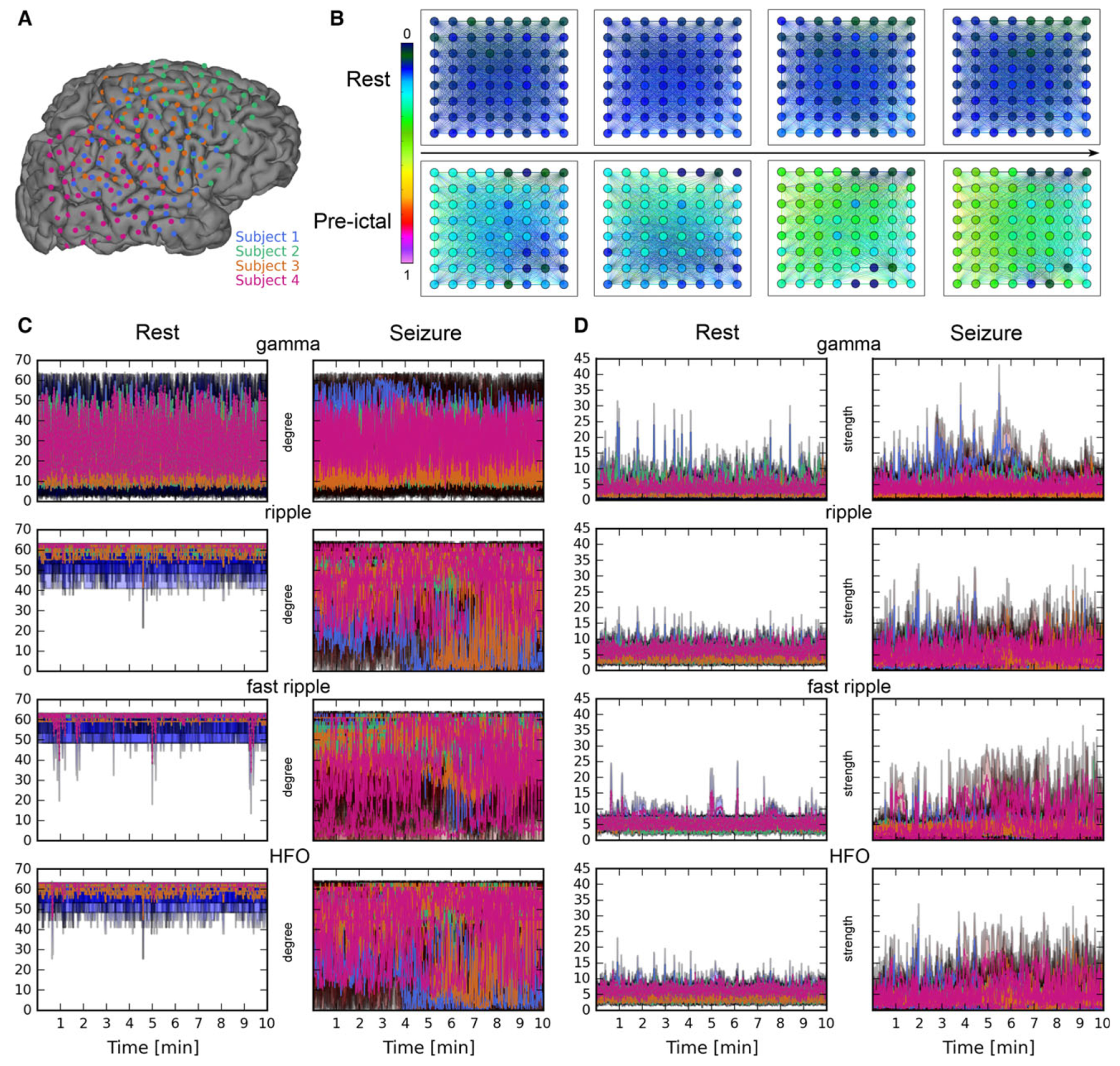
Spatiotemporal dynamics of cortical network activity during rest and seizure periods. (**A**) Representation of the subdural grid electrodes on the cortical surface in the standard Montreal Neurological Institute (MNI) space in each subject (patient) (color-coded). See individual grid placement map in Figure S1. (**B**) Representative HFO (100–500 Hz) functional network connectivity depicting 1-s rest and preictal windows, illustrating rapid changes of functional connectivity before the onset of an electrographic seizure compared to rest in patient 1. Edge and node color represent normalized mutual information coefficient and strength values (normalized between 0 and 1), respectively. Similar patterns were seen during other preictal periods across all four patients (Videos S1–S4). The arrow between the rest and preictal snapshots indicates the direction of time evolution. (**C**) Temporal dynamics of the mean degree during rest and seizure periods in gamma, ripple, fast-ripple, and HFO frequency range. Each color represents a patient (corresponding to the grids from Panel **A**), with shaded areas indicating standard deviations of mean values. (**D**) Temporal dynamics of the mean strength during rest and seizure periods showed increased absolute value and frequency of spiking in mean strength values in all frequency bands.

All patients underwent chronic, video-monitored subdural iEEG recordings for localization of SOZs prior to surgical resection. Eligibility of patients was established based on extensive preoperative screening criteria, including medical history, physical examination, scalp EEG recordings, and brain imaging (computed tomography [CT], magnetic resonance imaging [MRI], and positron emission tomography [PET]). Based on scalp EEG recordings, neuroimaging, and other semiologic characteristics, the seizure focus in all four patients was localized to the right fronto temporoparietal region before subdural grid and strip implantation (Figs. 1A and S1). All patients had complex partial seizures in addition to secondarily generalized, tonic, or simple partial seizures (Table S1). In all four patients, electrographic SOZs were found within the subdural electrode grid (Fig. S1).

All iEEG recordings were collected over a period of 1–2 weeks, during which each patient had at least four seizures. Antiseizure medications were either discontinued or significantly reduced in dose during chronic recordings. None of the patients had any other major neurologic (other than epilepsy) or psychiatric disorders. All but one patient remained seizure-free.

All patients provided written informed consent for participation in the study, which was approved by the institutional review boards of the Icahn School of Medicine at Mount Sinai and Thomas Jefferson University.

### Data acquisition

All iEEG data were obtained following placement of an 8 × 8 (64 electrodes) subdural grid and strip electrodes with the center-to-center distance of 10 mm (Integra, Plainsboro, NJ, U.S.A.). Each electrode had an exposed surface diameter of 2.5 mm. iEEG time series were recorded with a sampling rate of 1 kHz per channel (Nihon Kohden America, Irvine, CA, U.S.A.). All patients underwent MRI following implantation of the subdural electrodes. Using FreeSurfer 4.1 software, imaging data were transformed to the standard Montreal Neurological Institute (MNI) space, and a surface-based co-registration^12^ with the MNI305 average brain was performed to derive normalized spatial coordinates for each electrode before mapping onto a three-dimensional (3D) brain model (Fig. 1A). The electrodes’ coordinates were then transformed from patient’s native surface space onto the average pial surface.^13^ This method allowed concordant localization across patients.

### Data selection and preprocessing

Following the marking of all electrographic seizure-onset times based on iEEG and simultaneous video recordings in all patients by an experienced clinical neurophysiologist/epileptologist (MRS), a total of 24 resting (interictal) and 24 seizure periods, each 10 min in duration, were extracted for further processing. All resting periods were chosen with maximal temporal distance (2–48 h) from a clinical or electrographic seizure episode in each patient. In addition, a single 60-min interval (28 min before and 22 min after a 10-min seizure period harboring a 76-s electrographic seizure) was examined to assess the extended temporal evolution of HFO network dynamics.

The iEEG data from the entire 8 × 8 grid were bandpass filtered in four frequency bands: 30–80 Hz (gamma band), 80–250 Hz (ripple band), 250–500 Hz (fast ripple band), and 100–500 Hz (HFO band). A finite impulse-response Kaiser filter was used with 30-dB minimal-frequency attenuation in the stop-band, 30 dB maximal frequency loss in the pass-band, and 10 Hz lower/upper transition width.^14^ All data segments were forward/backward filtered to avoid phase distortions. Although lower and upper bounds of the filters were narrowed by transition width partially accounting for attenuation effects around the Nyquist frequency, the subsequent application of a high-frequency content detection algorithm before constructing functional entropy networks effectively erased any aliasing artifacts.

Next, ripple, fast ripple, and HFO-band filtered data were scanned for high-frequency events of interest (HFEoIs) based on a previously reported computational algorithm for HFEoI detection.^15^ This ensured maximal detection sensitivity for each channel’s HFO content to sustain the preprocessing pipeline. The subsequent statistical interaction analysis quantified the amount of shared high-frequency information per second between channels. HFEoIs were selected on the basis of the following steps (algorithmic details are provided in the Data S1, Supporting Information and Fig. S6):

1. A threshold was set at one standard deviation above each channel’s absolute mean signal.
2. A smooth approximate envelope was constructed by calculating the absolute value of the analytic representation of the thresholded signal and convolving it with a Gaussian kernel. The smoothing step was introduced as a computationally efficient means to merge regions with very short interevent intervals.
3. The average value of the smooth envelope over nonzero regions of the thresholded signal was then used to construct a smooth cutoff curve, which ensured that only events with a sufficient number of peaks above the given threshold were selected.
4. Signal segments corresponding to nonzero regions of the smooth cutoff were marked as HFEoI.

### Network construction and graph theoretical analysis

Functional connectivity derived from the pair-wise interactions of electrodes in the 8 × 8 grid was quantified by computing the normalized mutual information (NMI)^16^ between each pair of preprocessed 1-s long electrode time-courses. The NMI is a measure of statistical dependence, which assumes values between zero (statistical independence) and one (absolute mutual dependence), allowing for quantitative comparisons of NMI coefficients across different data sets. The calculated NMI values were used to construct matrices so that every second of iEEG data was represented by a 64 × 64 (8 × 8 grid = 64 electrodes) NMI matrix. In this manner, 28,800 NMI matrices were constructed, corresponding to 24 resting and 24 seizure periods of 10-min each. By interpreting electrodes as nodes and NMI coefficients as edge-weights, we constructed weighted undirected graphs with N = 64 nodes. Thus, the computed NMI networks provided a second-to-second snapshot of similarities in electrode activity across the entire grid.

The constructed graphs were analyzed using a “bottom-up” approach by quantifying the temporal patterns of local metrics of nodal influence (i.e., nodal degree and strength) followed by estimation of global features of the network architecture (i.e., average clustering coefficient), and finally an assessment of community dynamics, which allowed for detailed delineation and quantification of the spatiotemporal evolution of the network architecture. To quantify a single node’s importance within the network, its degree (i.e., the number of connected edges) and strength (i.e., the sum of connected edge weights) were computed. As a first approximation to quantify network segregation, the global clustering coefficient of each graph was calculated by averaging local clustering coefficient values, which were computed as the geometric mean of weights in triangles around each node in the network.^17^ Network segregation and integration were assessed by estimating the graph’s optimal modular decomposition.^18^ A network’s optimal modular partition divides the graph into connected components that show maximal number of within-group and minimal number of between-group edges. Optimal modular decompositions of per-second NMI networks were estimated using the following algorithmic strategy. As the initial step, each node was assigned a unique module number, that is, module 1 comprised node 1, module 2 comprised node 2, and so on, such that the initial community affiliation vector was M^0^ = (1,2,…,64). This module assignment was then used to initialize a community detection strategy based on the Kernighan-Lin algorithm,^19^ yielding a refined partition vector M^1^. Because the underlying optimization routine of the employed algorithm was based on random permutations of nodal group assignments, the community detection was repeated using M^1^ as the initial condition, which resulted in an updated partition vector M^2^. Altogether, community detection calculations were repeated 100 times per network to ensure stability of the computed partitions. The final community affiliation vector was obtained by computing the average network partition across all iterations. Thus, the resulting nodal community membership was based on how frequently a node was assigned to the same module. This algorithmic strategy was applied to every 1-s NMI network for all resting and seizure periods.

A linear mixed-effect model analysis^20^ was performed to assess the influence of frequency and physiologic state (rest, preictal, ictal, and postictal states) on network properties (i.e., degree, strength, clustering coefficient, and module count). Frequency and pathophysiologic states were entered as interacting fixed effects into the model. As random effects, we entered an intercept for patients and a by-patient random slope for the effect of the pathophysiologic state. Likelihood ratio tests were used to assess whether the full model with the pathophysiologic state as main effect was significantly different from a null model without the effect at a corrected p ≤ 0.05. Statistical significance of differences in network properties (i.e., nodal influence, clustering coefficient, and community structure) between the resting state and preictal, ictal, and postictal periods across all four patients was assessed using the fitted models in a many-to-one Dunnett contrast at p ≤ 0.05 adjusted for multiple comparisons.^21^

Modular decompositions were calculated in Matlab (MathWorks Inc., Natick, MA, U.S.A.) using the Brain Connectivity Toolbox.^22^ Statistical analysis of network properties was performed in R.^23^

## Results

A likelihood ratio test of the constructed linear mixed effect models showed that temporal variations of network structure were related to pathophysiologic states (all corrected p ≤ 0.0052).

The temporal evolution of nodal degrees within the gamma, ripple, fast-ripple, and HFO networks was characterized by stable resting-state (i.e., interictal) connectivity patterns (Fig. 1B, videos S1–S4). However, these patterns were significantly disrupted along the entire time course of seizure periods (Fig. 1B, videos S1–S4). Specifically, during seizures, high-frequency (ripple, fast ripple, and HFO) networks were characterized by distinct decreases in nodal degree (i.e., the number of connections) (all corrected p ≤ 0.006), whereas gamma-band networks did not show statistically significant differences in degree values between seizure and resting periods (all corrected p ≥ 0.9) (Table 1; Fig. 1C). Similarly, high-frequency networks but not gamma band networks exhibited significant increases in nodal strength (i.e., connection weights) during seizure periods as compared to the resting periods (all high-frequency networks vs. rest at a corrected p ≤ 0.003; gamma network vs. rest at a corrected p ≥ 0.8) (Table 1; Fig. 1D).

To estimate dynamical changes in network segregation, we assessed the temporal evolution of global clustering coefficients during seizure periods as compared to resting values. Before the onset of an electrographic seizure, preictal high-frequency networks (including ripple, fast ripple, and HFO networks) showed statistically significant differences in clustering coefficient compared to the resting state (all corrected p ≤ 0.03, Table 1). On the other hand, gamma networks exhibited pronounced increases in global clustering only during the ictal period (corrected p = 0.01) (Table 1). No statistical differences in clustering coefficient were found between resting state and the postictal periods at any frequency band (p ≥ 0.3).

**Figure 2.**
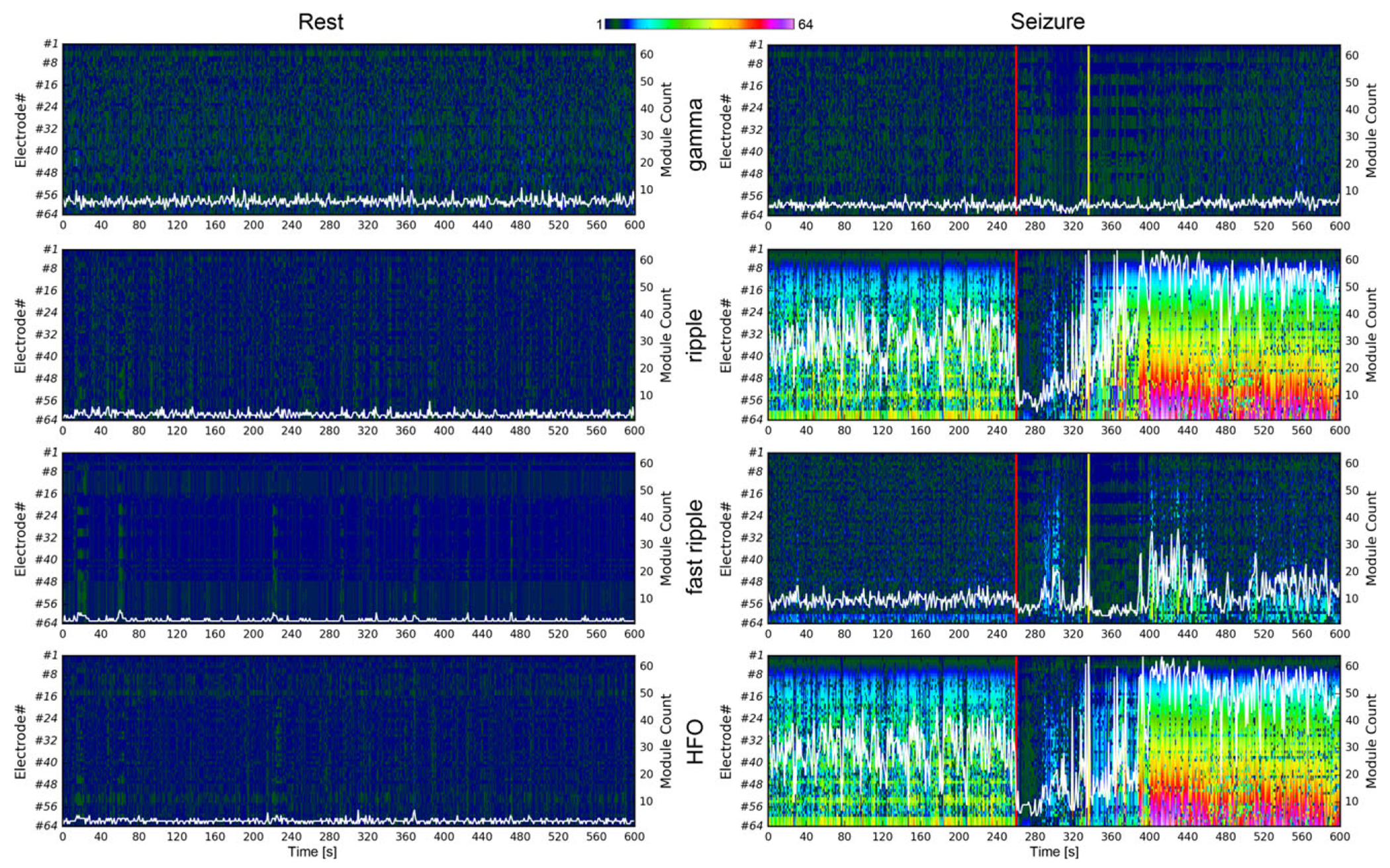
Functional cortical network architecture at different frequency bands at rest and during a seizure period. The spatiotemporal evolution of the modular structure of functional cortical networks in gamma, ripple, fast-ripple, and HFO frequency bands in patient 1 during 600-s-long rest (left column) and seizure periods (right column). Each node is assigned a number in the left vertical axis, and each community of nodes is represented by a color. Starting with electrode 1 at the top, all the nodes in a network are shown as a column of pixels. The dimension of each network is N = 64, hence each column is 64 pixels long with each pixel shaded using a 6-bit (2^6^ = 64) color palette, representing the maximal possible number of modules. In this manner, all networks are represented by 6-bit pixel columns, which are stacked against each other, starting from the left and advancing in one-second steps in the time dimension to 600. Red vertical lines mark the electrographic seizure onset; yellow vertical lines highlight the end of the ictal period. The overlaid white curves demonstrate the stable temporal evolution of the number of network modules (right axes) during the resting periods in all frequency bands, but their significant deviations from resting period values during a seizure period in all high-frequency bands. Similar patterns were observed in all other patients.

**Figure 3.**
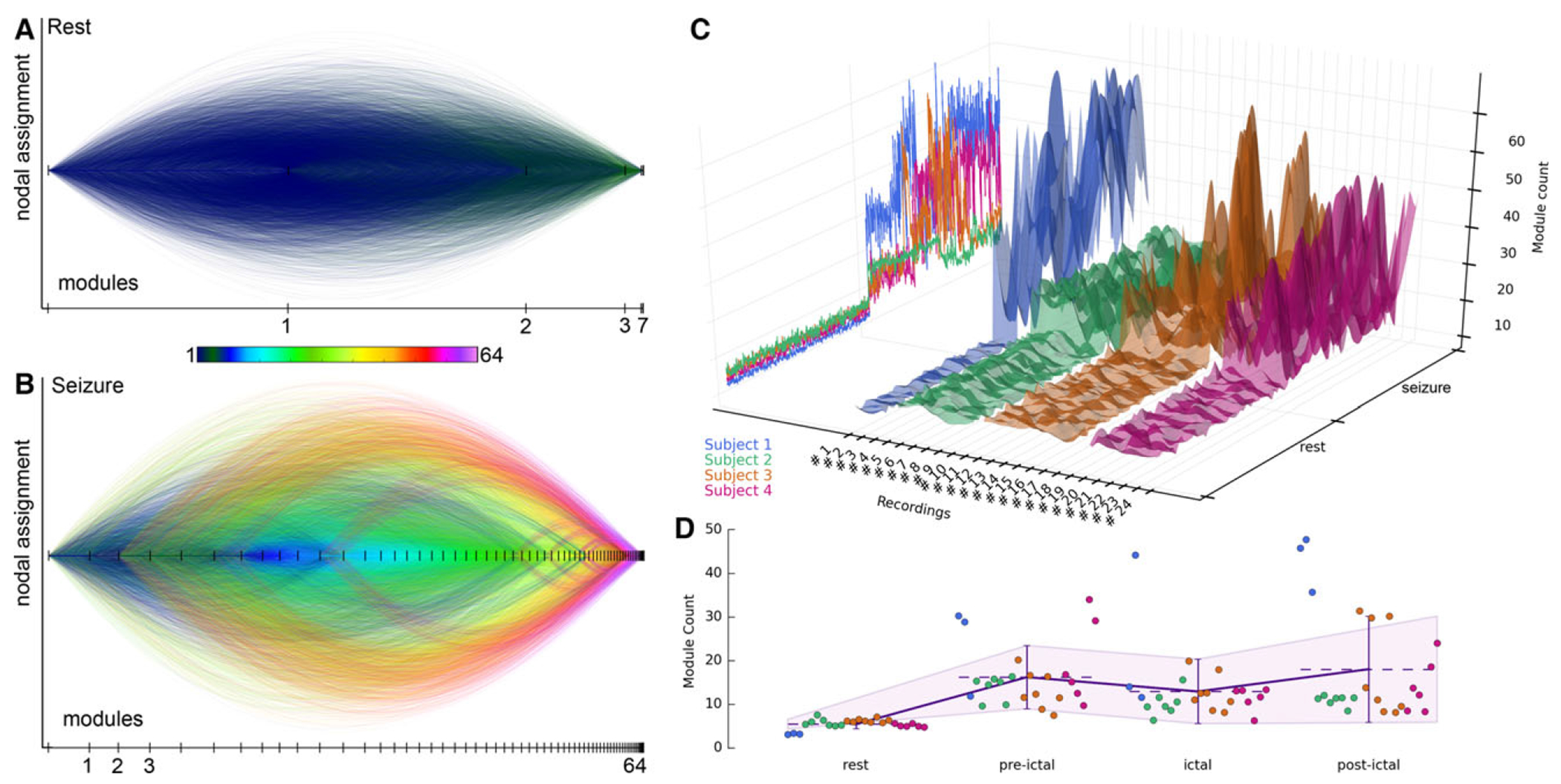
Modular disorganization of functional HFO networks during seizure periods leads to the emergence of numerous small functional communities. (**A, B**) The horizontal axis represents the total number of network modules emerging during a rest (**A**) and seizure period (**B**) in patient 1 (same periods as shown in Figs. 2 and 4) with the length of each line segment indicating the corresponding module size. Color-coded arcs mark the peculiar dynamics of the modular affiliation of each node with the line color indicating the target module. At rest, three dominant modules comprised the network; however, during the seizure period, numerous small modules fragmented the HFO networks (i.e., modular breakdown). Similar patterns were observed in all other patients (Fig. S5). (**C**) Temporal evolution of the module count in HFO networks across all analyzed rest and seizure periods (1–24). Raw unsmoothed data is shown as relief contour in the left vertical plane. (**D**) Representation of module count averaged across all functional HFO networks during all 24 resting and 24 seizure periods (rest, preictal, ictal, and postictal phases), color-coded for each patient. Dashed lines show the mean module count averaged across all recordings and patients, with vertical error bars representing standard deviations (Table 1).

To further quantify these pathologic alterations in network architecture during the transition from rest to preictal to ictal and postictal periods, we used the concept of network modules, that is, the formation of nodal communities based on their shared activity (see Methods). During the resting state, networks at all frequency bands were characterized by a stable modular structure, with an average number of modules ranging from 3.3 to 7.2 (Table 1). Gamma networks retained the community structure of the resting state through the entire length of a seizure period (Fig. 2) and did not show significant changes in the number of modules between resting and seizure periods (corrected p = 1.0) (Table 1). Conversely, significantly irregular partitioning of the network architecture with an increased average number of modules (ranging from 22.7 to 29.4) during the preictal period was observed across all high-frequency networks, including ripple, fast-ripple, and HFO networks (all corrected p ≤ 0.03) (Table 1). This effect was associated with an increased migration of nodes between modules in the high frequency band over the entire time course of a seizure (number of modules transitioned by each node during rest vs. seizure period in the ripple band: 13.9 ± 1.9 vs. 33.4 ± 6.6; fast ripple band: 8.6 ± 1.6 vs. 23.6 ± 5.2; HFO: 12.0 ± 1.8 vs. 34.1 ± 7.3) (Table 2, Figs. 3A,B and S5).

As a result, in the high frequency bands, the highest number of modules was formed during the pre- and postictal periods and the lowest number of modules was formed during the ictal periods (Table 1, Fig. 3C,D). The fragmentation of the network into numerous small modules ultimately resulted in a breakdown of its architecture. This modular breakdown was seen on average 225 s before the electrographic seizure onset (ripple band: 241.5 ± 114.0 s; fast ripple band: 166.5 ± 124.5 s; HFO band: 225.0 ± 106.5 s) and 190 s after the termination of seizures (ripple band: 175.0 ± 121.5 s; fast ripple band: 193.5 ± 105.0 s; HFO band: 190.5 ± 114.0 s) (Table 2).

**Figure 4.**
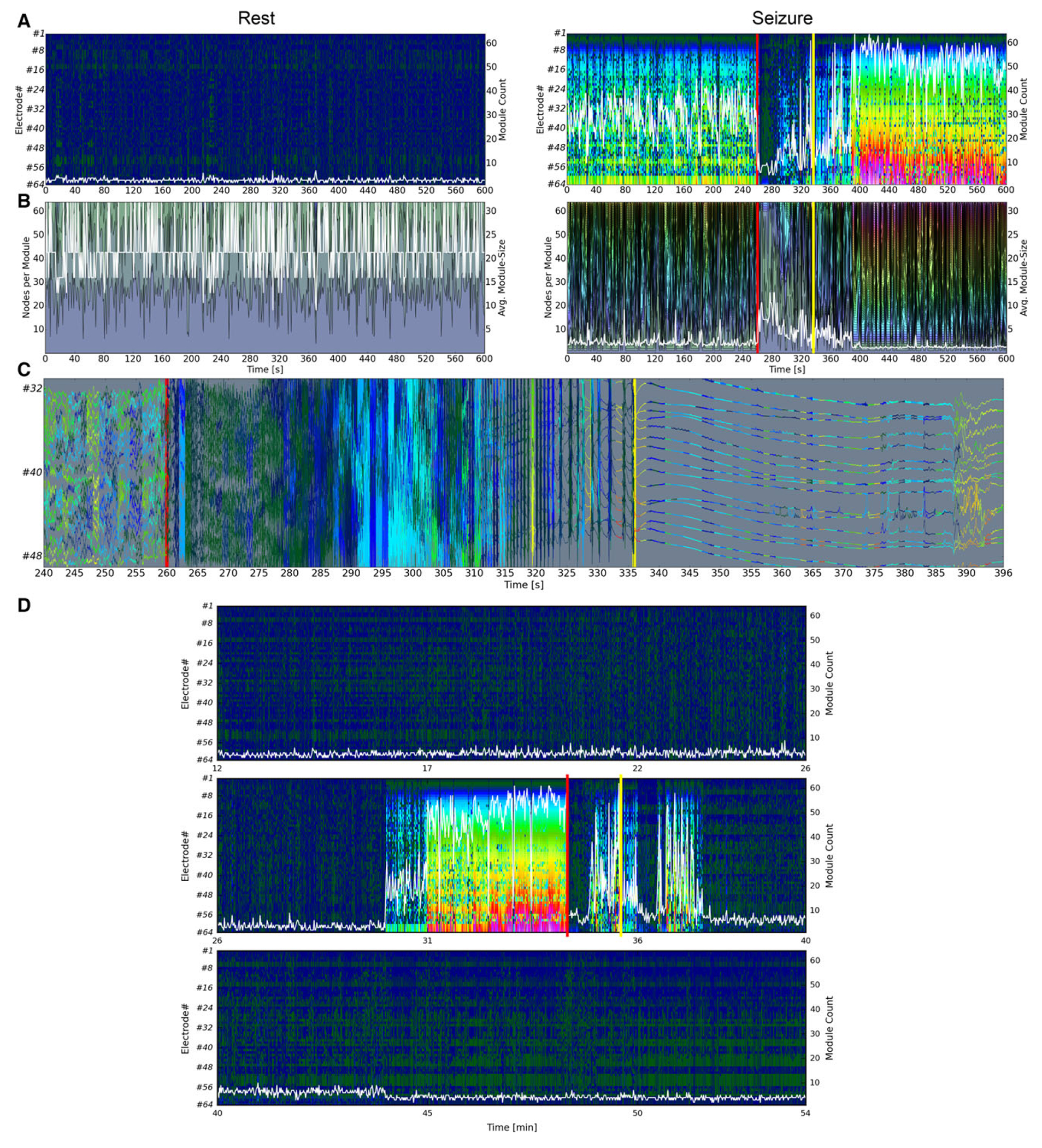
Preictal, ictal, and postictal functional HFO networks undergo similar patterns of modular breakdown despite widely contrasting electrographic patterns. (**A**) The spatiotemporal evolution of modules in functional HFO networks at rest (left column) and during a seizure period (right column) in patient 1. Each row corresponds to a node (1–64), and each column represents the whole network at a specific time point. Nodes within the same community share the same color. Red vertical lines mark the electrographic seizure onset; yellow vertical lines highlight the end of the ictal period. The overlaid white curves demonstrate the stable temporal evolution of the number of network modules (right axes) during the resting period but their significant deviations from resting period values over the time course of a seizure period. (**B**) Spatiotemporal evolution of module size in functional HFO networks in the same patient during the same resting and seizure periods. Each color represents a module with the vertical extension of each shaded area indicating its size (the current number of nodes in that module). The overlaid white curve shows the mean (averaged across the network) module size (right axis). The resting state is characterized by the prevalence of a few large modules that will subsequently fragment into numerous small modules during the seizure period. (**C**) The corresponding unfiltered 16-channel iEEG segment (electrode 32–48) illustrates a short preictal period followed by ictal onset and termination (red and yellow lines) and a short postictal period (color-coded based on modular affiliations of the corresponding nodes in the functional HFO networks). Note the widely different electrographic patterns of cortical activity before the seizure onset (segment before the red line), during the electrographic seizure (segment between the red and yellow line), and after seizure termination (segment after the yellow line), and yet their similar modular pattern as illustrated in **A** and **B** (increase in module count and corresponding decrease in module size compared to resting period values) (Table 1). Similar patterns were observed in all other patients during rest and seizure periods (Figs. S2–S4). (**D**) A 42-min segment of iEEG was analyzed to show the continuous spatiotemporal evolution of the modular structure from its resting pattern, throughout an entire seizure period and its return to the resting pattern in patient 1. The overlaid white curves demonstrate the stable temporal evolution of the number of network modules (right axes) during the resting (interictal) period (upper and lower panels) but their significant deviations from resting period values throughout the time course of the preictal, ictal, and postictal periods collectivley represented as seizure period (middle panel). To account for the extended time interval, HFEoI detection was performed using a moving average/standard deviation with a window size of 1 min.

Ripple and fast ripple networks showed competing community patterns during seizure periods; modular disorganization was observed in either the ripple or fast ripple band but not in both simultaneously, whereas HFO networks exhibited an abnormal architecture that combined features of both ripple and fast-ripple networks (Fig. 2). The three salient phases of the seizure period—preictal, ictal, and postictal periods—undergo similar patterns of modular disorganization (increase in number of modules and decrease in module size, Figs. 4A,B and S2–S4) despite their widely contrasting electrographic patterns shown in the unfiltered 16-channel iEEG (Fig. 4C). Taken together, these results point to pronounced variations in nodal connectivity patterns (i.e., NMI coefficient values) that initiate before electrographic seizure onset and is suggestive of the existence of a state of high ictogenic potential.

Analysis of an extended segment of iEEG that included the spatiotemporal evolution of the modular structure of HFO network from resting to seizure to resting periods showed the emergence of a highly dynamic state of modular disorganization, that was characterized by numerous small modules more than 3 min prior to the electrographic seizure onset (Fig. 4D, middle row). This state was surrounded on each side by presumably resting-like periods with stable modular patterns (reminiscent of the resting period networks in Fig. 2) before and after the seizure period (Fig. 4D, top and bottom panels).

## Discussion

In this study, we used iEEG recordings in patients with intractable epilepsy to demonstrate unique changes in the modular organization of high-frequency cortical networks prior to electrographic seizure onset. Abnormalities in the number and strength of network connections led to pathologic segregation of the entire recorded network during the preictal state on average 225 s before the electrographic seizure onset, which led to the breakdown of the modular network structure across the entire seizure period and extended on average 190 s into the postictal period (Table 2). Furthermore, this modular breakdown was not limited to seizure focus alone but rather engulfed larger neocortical areas (within the confines of the subdural grid), likely representing a dynamic state with heightened ictogenic potential facilitating seizure initiation and spread.^24^

One of the prominent features of the pre- and postictal periods was spatiotemporally rapid de- and resynchronization of high-frequency cortical activity, which caused continuously changing modular affiliations of nodes and modular instability in the high-frequency networks. In contrast, increased synchrony at ictal onset led to a sudden spike in the connectivity profile (degree/strength) of nodes, which formed a few large but highly unstable modules. Such a rapid buildup and breakdown of network synchronization among nodal communities was not observed during the resting period in any frequency band. Taken together, our data indicate that aberrant topology and modular breakdown of high-frequency networks are a hallmark of an epileptic seizure, evolving before its electrographic onset and undergoing gradual normalization of network architecture in the postictal period.

When examining different frequency bands within the high-frequency spectrum, we found that ripple or fast-ripple networks shaped the HFO network community structure, and as such, the paroxysmal modular breakdown characteristic of HFO networks during seizure periods did not seem to be balanced between the ripple and fast ripple components. This suggests that HFO networks represented an amalgamation of ripple and fast-ripple networks, reflecting the dynamic network changes of both frequency bands. Taken together, these findings underscore the importance of analyzing the functional connectome of seizures in a broad band (ripple and fast ripple) high-frequency spectrum.

Although the cellular and synaptic mechanisms of HFO remain partially understood, it has been shown that correlated neuronal activity through the activation of axonal plexus may underlie the generation of HFOs^25,26^ recorded across relatively large areas of cortex. These high-frequency local field potentials (LFPs) are generated primarily as HFOs in the principal cell axonal plexus,^4,27,28^ may manifest as spikelets and full or partial spikes in the soma of principal cells^4,5,9,25^ and lead to highly synchronized high-frequency band activity in cortical sites that are often tens of millimeters apart from each other.^9,29,30^ Nonsynaptic (gap junctional) mechanisms have been proposed as a potential process underlying the propagation of spikes (a term more akin to a partial or complete axonal action potential and different from the term used in clinical epilepsy, e.g., interictal spikes) among sparsely connected cortical principal neurons at a specific subcellular compartment, that is, axons that give rise to coherent oscillations in principal cell axonal plexus.^4,31,32^ An axonal plexus formed by strong yet sparse (with the network connectivity above the percolation threshold) electrical coupling via gap junctions has been shown to shape the random, unstructured spontaneous activity of the plexus into coherent and self-organized oscillations under certain conditions.^31,32^ In fact, the frequency of such oscillations does not depend on intrinsic properties of the principal cells, or the network size (if sufficiently large), but mainly on the rate of axonal spontaneous activity and the topology of the network.^31–33^ In line with these earlier findings, our current iEEG-based data demonstrated significant local and global abnormalities of high-frequency networks, which were not confined to the seizure foci but spread and encompassed the larger recorded network. As shown in other studies, synchronization across such large areas of cortex is unlikely to be orchestrated by multiple LFP sources generated by uncorrelated synaptic activity or local field effects.^34^ In support of this, we have shown that pathologic HFO networks have similar breakdown patterns across multiple seizure types (complex partial, generalized tonic–clonic, and secondarily generalized tonic–clonic) and therefore, likely manifest a common underlying epileptogenic process.

This nonsynaptic mechanism is distinctly different from other well-described modes of generation of oscillatory activity, where intrinsic oscillatory dynamics of individual neurons coupled by gap junctions organize coherent oscillations, such as gamma oscillations.^35^ In our study, gamma networks showed elevated average clustering coefficients during the ictal period only as compared to the resting period, which supports the notion of increased local synchronization shown by other investigators.^36^ However, the modular decomposition of gamma networks was characterized by a transient change in community structure only during the ictal but not in the pre- or postictal periods. In other words, gamma networks exhibited changes in their modular structures only after the full manifestation of the epileptogenic process (i.e., only during the ictus) and not in the preictal period.

Furthermore, the HFO network dynamics were found to be significantly different from the gamma network dynamics during the seizure period, which is suggestive of the fundamental differences in the generation of these two oscillations at the cellular and network levels. In contrast to gamma oscillations, the cellular mechanisms underlying HFOs do not involve chemical synaptic transmission, as its blockage among excitatory and inhibitory cortical neurons eliminates all epileptiform discharges (e.g., sharp waves and spikes), except HFO.^4,26^ More specifically, neither reduction nor complete blockade of γ-aminobutyric acid (GABA)ergic synaptic transmission affects HFO. Similarly, HFOs persist under conditions of near complete elimination of interneuron gap junctions in connexin36-knockout mice, while gamma oscillations dramatically decrease.^37^ It is, therefore, plausible that gamma and HFO networks play distinct roles in epileptogenesis.

We found that modular disorganization of HFO networks precedes electrographic seizures and persists through the ictal and postictal periods. This modular disorganization is based on cortical HFOs that are mostly if not exclusively generated through nonsynaptic mechanisms between cortical principal neurons and therefore are independent of synaptic mechanisms driving the observed electrographic seizure activity such as spikes, sharp waves, and most if not all the slow and fast cortical oscillatory activity up to and including the gamma oscillations. In this sense, the spatiotemporal maps of HFO networks as shown in this study may be regarded as nonsynaptic biomarkers of ictogenesis that drive a network of cortical neurons connected via chemical synapses, ultimately generating epileptic seizure activity.

Our statistically significant findings demonstrate abnormal local nodal community structure and modular breakdown of high-frequency networks across all seizure events, which is consistent with the epileptogenic potential of HFOs independent of the clinical seizure presentation.^1^ As such, *pathologic HFO network dynamics* are likely to play a prominent role in human epileptogenesis. Therefore, measures of HFO network dynamics in contrast to single channel pathologic HFO^38,39^ have a high potential in facilitating the development of novel biomarkers for epileptogenesis. Future studies should take advantage of this specific network marker for temporal prediction of seizures and possible intervention via a closed loop (e.g., responsive) stimulation. Taken together, our study provides strong experimental evidence for the crucial role of high-frequency network oscillations in the transition from an interictal to a preictal state where further evolution to an ictal state may be inevitable.

## Supplementary Material

SFig.1 **Figure S1.** 3D reconstruction of intracranial (subdural) grid and strip electrode placement maps in all patients.

SFig.2 **Figure S2.** Functional cortical network structure at different frequency bands during rest and seizure periods for patient 2.

SFig.3 **Figure S3**. Functional cortical network structure at different frequency bands during rest and seizure periods for patient 3.

SFig.4 **Figure S4.** Functional cortical network structure at different frequency bands during rest and seizure periods for patient 4.

SFig.5 **Figure S5.** Modular affiliation dynamics in functional HFO networks at rest and during seizure periods.

SFig.6 **Figure S6.** Community maps based on Burnos HFEoI detection.

SMovie1 **Video S1.** Spatiotemporal evolution of functional HFO (100–500 Hz) networks during a representative resting (left) and seizure period (right) in patient 1.

SMovie2 **Video S2.** Spatiotemporal evolution of functional HFO (100–500 Hz) networks during a representative resting (left) and seizure period (right) in patient 2.

SMovie3 **Video S3**. Spatiotemporal evolution of functional HFO (100–500 Hz) networks during a representative resting (left) and seizure period (right) in patient 3.

SMovie4 **Video S4**. Spatiotemporal evolution of functional HFO (100–500 Hz) networks during a representative resting (left) and seizure period (right) in patient 4.

Supporting Material **Data S1.** Methods.
**Table S1.** Clinicopathologic characteristics of patients with drug-resistant epilepsy.

Additional Supporting Information may be found in the online version of this article:

## Figures and Tables

**Table 1.** Cortical network properties during resting and seizure periods

Frequency band	Network metric	Pathophysiologic state						
		Rest	Preictal	p-Value	Ictal	p-Value	Postictal	p-Value
Gamma	Degree	21.7 ± 7.4	22.8 ± 10.3	1.0	22.5 ± 10.2	1.0	23.6 ± 10.0	0.9
	Strength	3.0 ± 1.0	3.3 ± 1.5	0.9	3.1 ± 1.3	1.0	3.5 ± 1.6	0.8
	Clustering	0.1 ± 0.03	0.1 ± 0.02	0.9	0.13 ± 0.04*	0.01*	0.11 ± 0.03	0.9
	Module no.	5.8 ± 1.0	5.6 ± 0.9	1.0	5.7 ± 0.9	0.9	5.7 ± 0.8	1.0
Ripple	Degree	62.1 ± 1.6	45.1 ± 9.8*	0.004*	44.9 ± 9.8*	0.005*	44.8 ± 9.7*	0.005*
	Strength	5.4 ± 1.2	3.5 ± 1.2*	0.0001*	3.4 ± 1.2*	0.0002*	3.6 ± 1.1*	0.0003*
	Clustering	0.08 ± 0.02	0.06 ± 0.02*	0.02*	0.08 ± 0.02	1.0	0.06 ± 0.03	0.4
	Module no.	5.9 ± 1.3	16.1 ± 6.6 *	0.03*	13.8 ± 9.3	0.08	19.4 ± 13.7	0.07
Fast ripple	Degree	62.4 ± 1.2	43.6 ± 15.1*	0.002*	42.6 ± 16.3*	0.002*	43.6 ± 15.2*	0.003*
	Strength	5.6 ± 0.7	2.9 ± 1.1*	0.0001*	2.7 ± 1.2*	0.0001*	3.0 ± 1.2*	0.0001*
	Clustering	0.09 ± 0.01	0.05 ± 0.02*	0.001*	0.08 ± 0.03	0.9	0.06 ± 0.03	0.3
	Module no.	4.3 ± 1.0	17.4 ± 12.0*	0.01*	11.7 ± 4.1	0.09	14.3 ± 6.6	0.2
HFO	Degree	62.4 ± 1.2	45.2 ± 10.7*	0.004*	44.7 ± 10.7*	0.004*	44.8 ± 10.5*	0.005*
	Strength	5.3 ± 1.2	3.2 ± 1.0*	0.0001*	3.0 ± 0.8*	0.0001*	3.3 ± 0.9*	0.0001*
	Clustering	0.08 ± 0.02	0.05 ± 0.02*	0.008*	0.08 ± 0.03	0.9	0.06 ± 0.03	0.4
	Module no.	5.4 ± 1.1	16.2 ± 7.3*	0.03*	12.9 ± 7.4	0.09	18.0 ± 12.2	0.09

All values were averaged across patients and the respective temporal segments during rest, preictal, ictal, and postictal periods. The average values (mean ± standard deviation) of the degree (average number of connections in the network), strength (sum of connected edge weights averaged across the network), global clustering coefficient (average local clustering coefficient), and module count are shown for each physiologic state.

Asterisk (*) indicates statistically significant differences in network measures compared to resting values across all four patients at p ≤ 0.05 (corrected for multiple comparisons using a Dunnett contrast).

**Table 2.** Gamma and high-frequency modular dynamics of cortical networks during seizure periods

Frequency band	patient no.	Modular characteristics			
		No. of modules transitioned by a node		Timing of modular breakdown (s)	
		Rest	Seizure period	Before seizure	After seizure
Gamma	1	17.1 ± 0.4	14.3 ± 0.9	n/a	n/a
	2	13.6 ± 2.1	12.6 ± 1.6	n/a	n/a
	3	17.7 ± 2.1	17.0 ± 2.2	n/a	n/a
	4	11.4 ± 5.8	12.7 ± 1.5	n/a	n/a
	Mean ± SD	**15.0** ± **3.0**	**10.9** ± **6.7**	n/a	n/a
Ripple	1	11.3 ± 2.6	58.0 ± 4.1	222 ± 72	294 ± 66
	2	15.9 ± 4.1	19.1 ± 1.5	246 ± 96	192 ± 132
	3	14.3 ± 2.1	29.2 ± 11.6	234 ± 114	126 ± 144
	4	14.1 ± 1.2	27.2 ± 9.0	264 ± 174	90 ± 144
	Mean ± SD	**13.9** ± **1.9**	**33.4** ± **6.6**	**241.5** ± **114.0**	**175.0** ± **121.5**
Fast ripple	1	4.9 ± 2.0	23.3 ± 5.9	72 ± 126	222 ± 60
	2	9.4 ± 1.4	17.7 ± 2.8	174 ± 144	234 ± 108
	3	8.3 ± 1.2	17.5 ± 7.8	90 ± 126	126 ± 126
	4	11.6 ± 1.8	35.7 ± 4.4	330 ± 102	192 ± 126
	Mean ± SD	**8.6** ± **1.6**	**23.6** ± **5.2**	**166.5** ± **124.5**	**193.5** ± **105.0**
HFO	1	8.1 ± 1.1	59.3 ± 4.6	222 ± 72	294 ± 66
	2	14.4 ± 2.9	18.9 ± 1.4	222 ± 126	210 ± 108
	3	12.4 ± 2.4	25.7 ± 12.1	138 ± 120	126 ± 144
	4	13.1 ± 0.8	32.3 ± 11.1	318 ± 108	132 ± 138
	Mean ± SD	**12.0** ± **1.8**	**34.1** ± **7.3**	**225.0** ± **106.5**	**190.5** ± **114.0**

The hallmark of modular breakdown is rapid transitions in modular affiliation of each node. The number of modules transitioned by each node at each frequency band during resting and seizure periods is shown as mean ± standard deviation in each patient, with the group mean ± standard deviation values shown in bold. Note the increase in the number of modules transitioned by each node during the seizure period compared to resting period in all high frequency bands. The modular breakdown precedes all electrographic seizure onsets in all patients and persists for several minutes after electrographic seizure termination. Modular breakdown was defined as a statistically significant increase in the number of network modules above the group-averaged resting values for each frequency band given in Table 1. SD, standard deviation; n/a, not applicable.

## Key Points

- Dynamics of cortical HFO networks in the preictal period are drastically different from those observed in the interictal period
- Cortical HFO networks undergo modular breakdown before electrographic seizure onset
- Modular breakdown of high-frequency cortical networks represents a state of high propensity to generate seizures and hence a network-wide biomarker of epileptogenesis
