# Supplementary figures and images for: High-frequency brain networks undergo modular breakdown during epileptic seizures
Source: Epilepsia. Author manuscript; Available in PMC 2026 Feb 6. (PMC12880085; doi:10.1111/epi.13413)

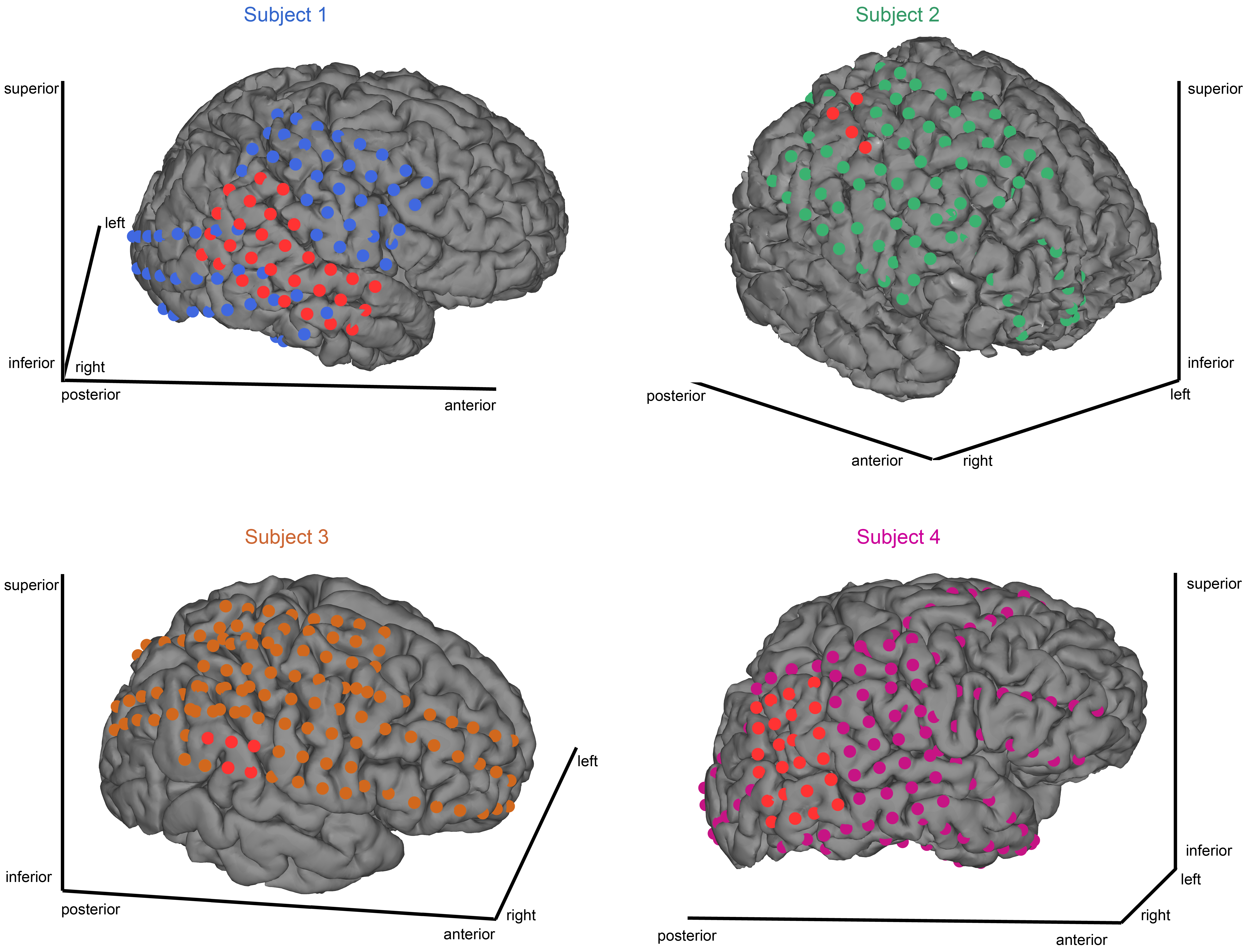

Supplement: SFig.1 — Figure S1. 3D reconstruction of intracranial (subdural) grid and strip electrode placement maps in all patients. [file NIHMS2136806-supplement-SFig_1.tif]

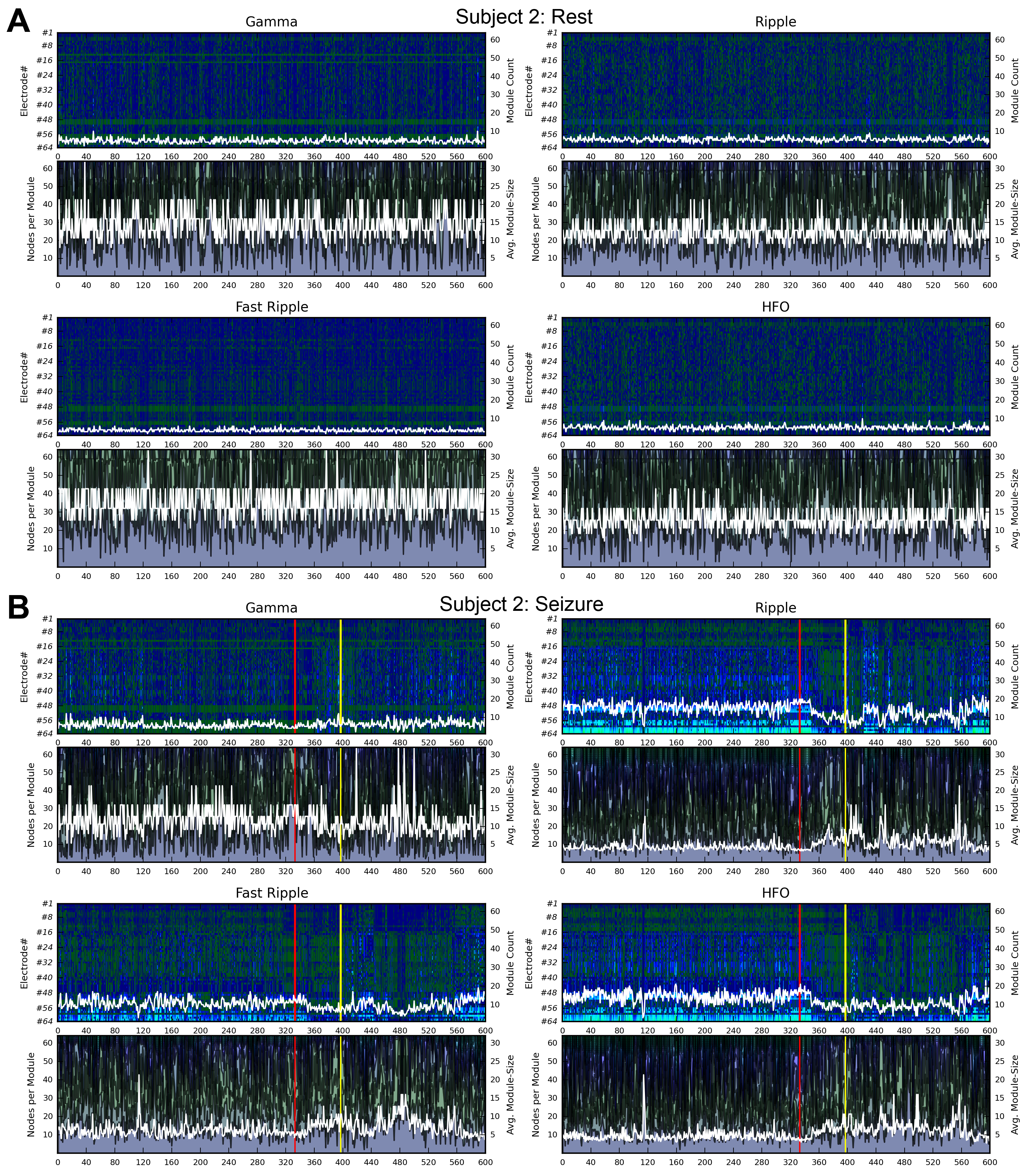

Supplement: SFig.2 — Figure S2. Functional cortical network structure at different frequency bands during rest and seizure periods for patient 2. [file NIHMS2136806-supplement-SFig_2.tif]

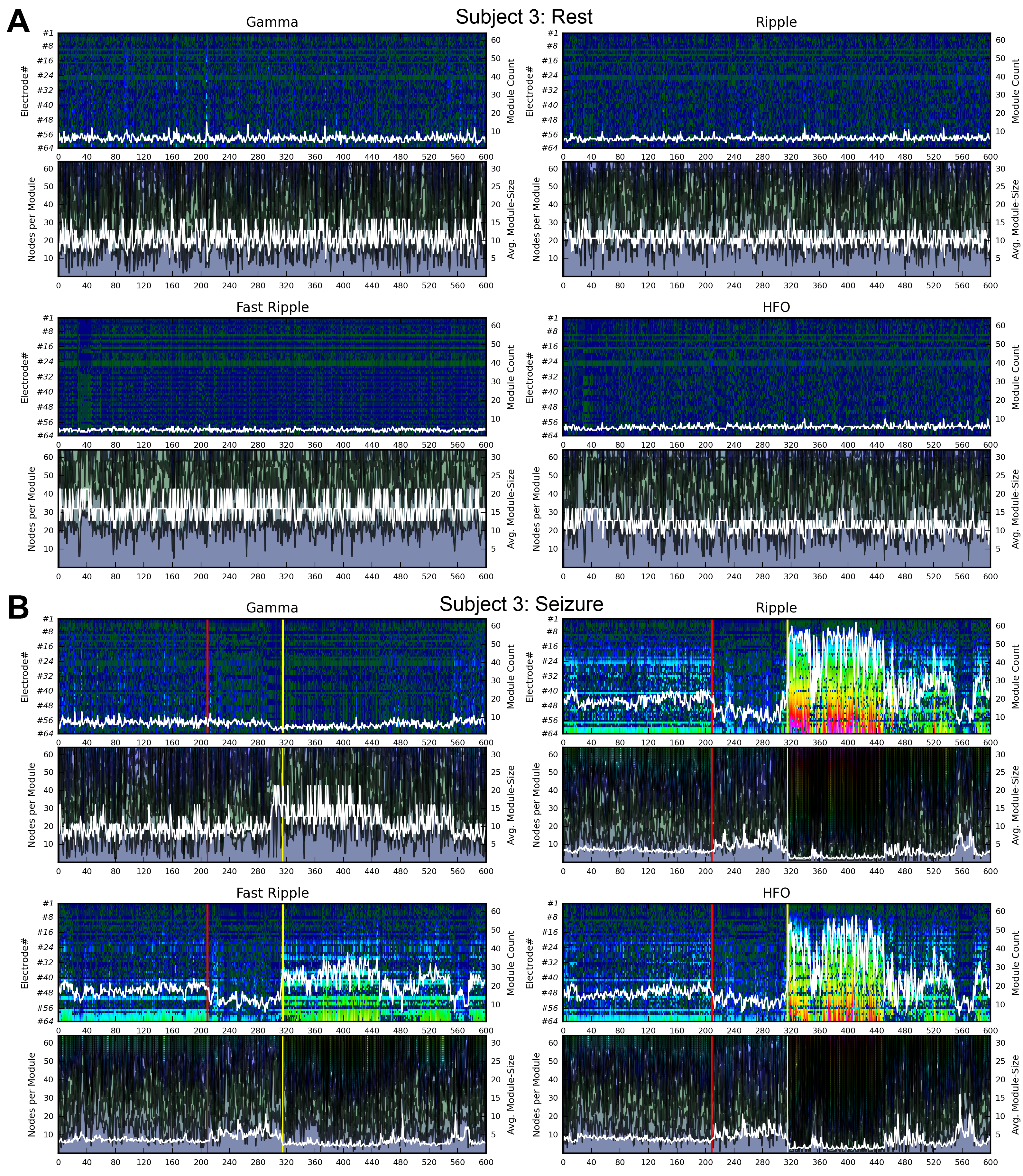

Supplement: SFig.3 — Figure S3. Functional cortical network structure at different frequency bands during rest and seizure periods for patient 3. [file NIHMS2136806-supplement-SFig_3.tif]

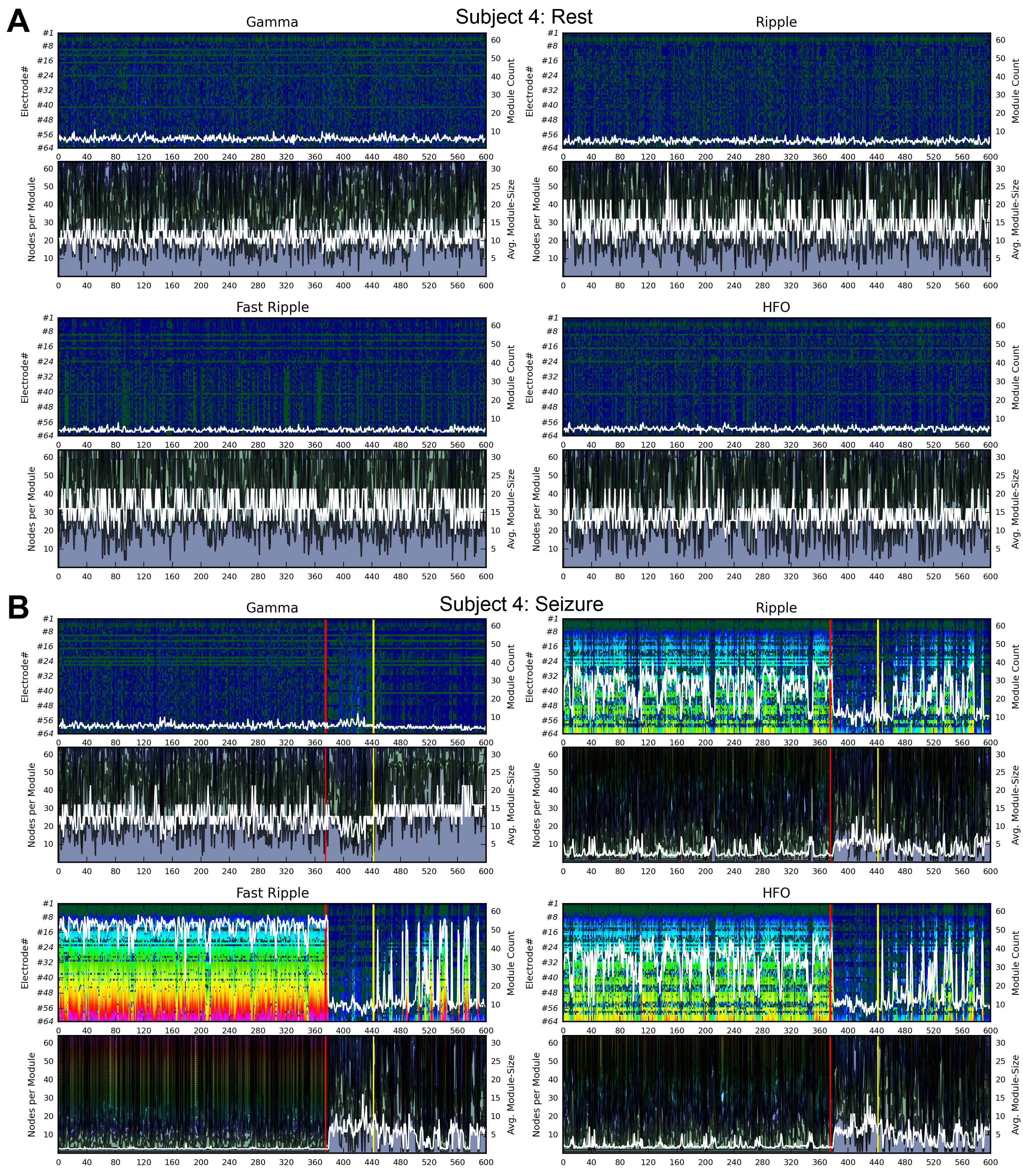

Supplement: SFig.4 — Figure S4. Functional cortical network structure at different frequency bands during rest and seizure periods for patient 4. [file NIHMS2136806-supplement-SFig_4.tif]

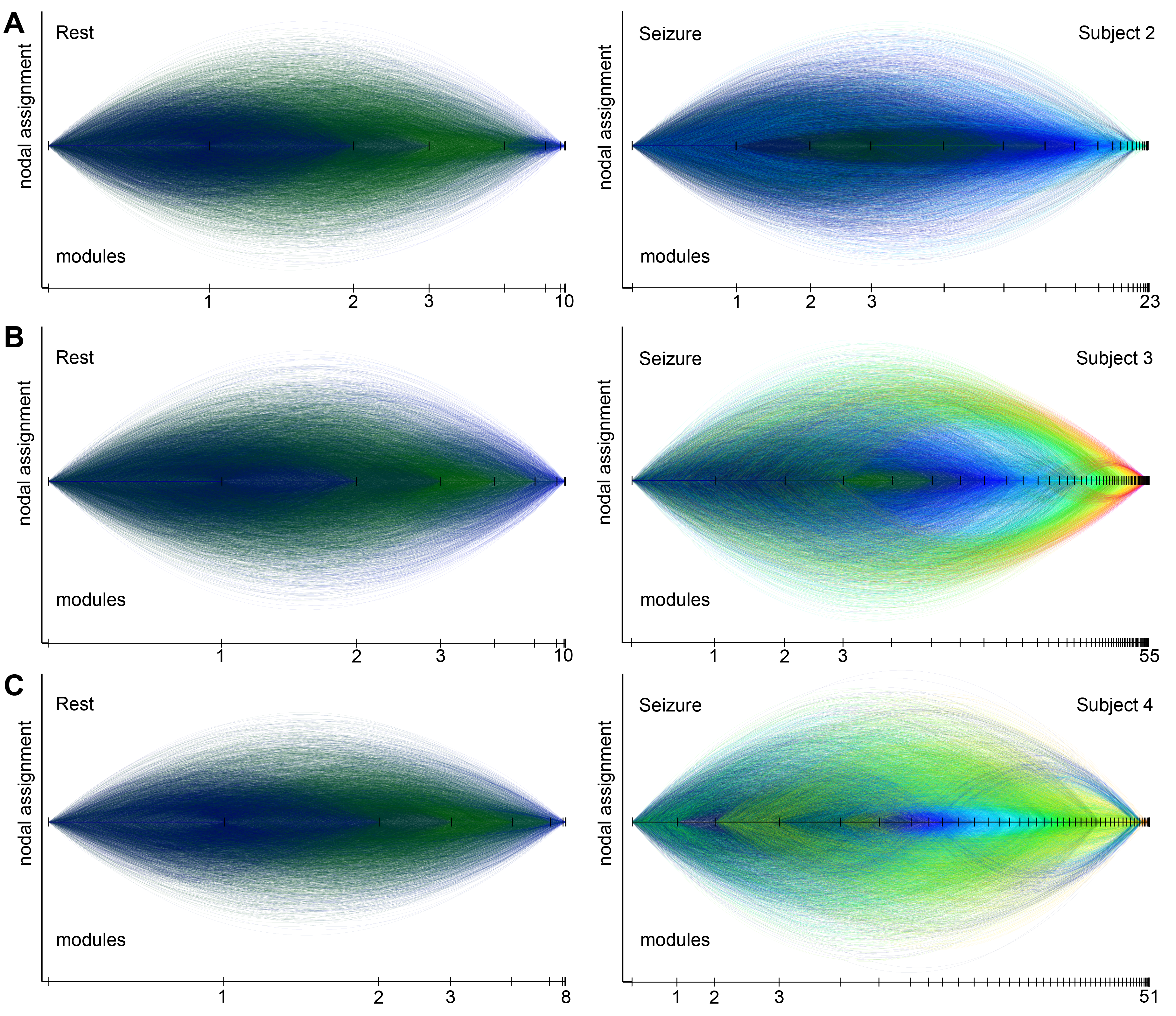

Supplement: SFig.5 — Figure S5. Modular affiliation dynamics in functional HFO networks at rest and during seizure periods. [file NIHMS2136806-supplement-SFig_5.tif]

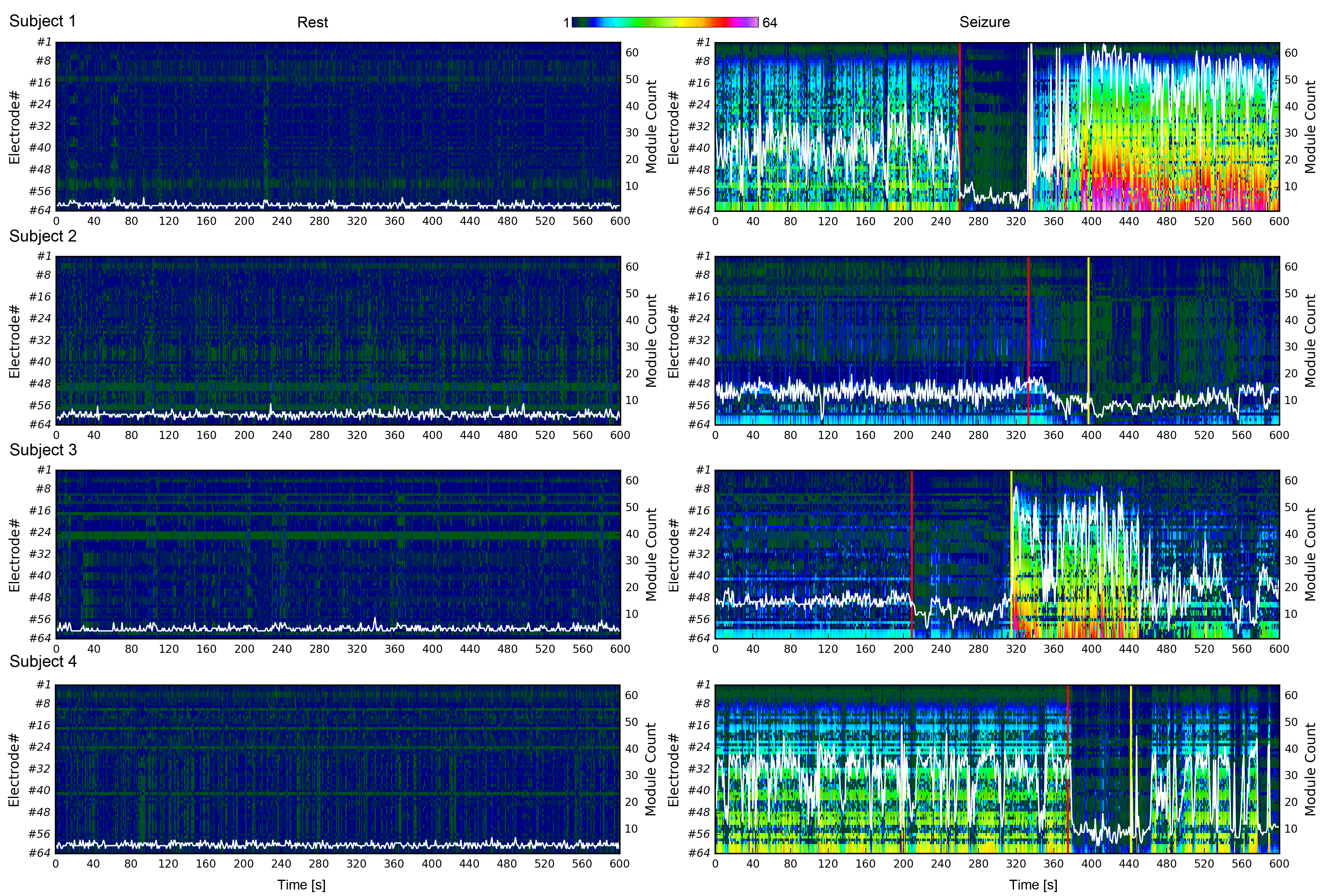

Supplement: SFig.6 — Figure S6. Community maps based on Burnos HFEoI detection. [file NIHMS2136806-supplement-SFig_6.tif]
