# Supplementary material for: High-frequency brain networks undergo modular breakdown during epileptic seizures
Source: Epilepsia. Author manuscript; Available in PMC 2026 Feb 6. (PMC12880085; doi:10.1111/epi.13413)
Supplement: Supporting Material — Data S1. Methods. Table S1. Clinicopathologic characteristics of patients with drug-resistant epilepsy. [file NIHMS2136806-supplement-Supporting_Material.doc]

Supporting Information

**High frequency brain networks undergo modular breakdown during epileptic seizures**

Stefan Fuertinger, PhD^1^, Kristina Simonyan, MD, PhD^1,2^, Michael R. Sperling, MD^3^,

Ashwini D. Sharan, MD^4^, Farid Hamzei-Sichani, MD, PhD ^4.5^*

**Methods**

*Subjects*

Seizure focus in all four patients was localized to the right fronto-temporo-parietal region based on evaluation of scalp EEG recordings, neuroimaging data, and other semiological characteristics before intracranial electrodes were implanted (Fig. S1).

*Figure S1 about here*

*HFEoI Detection Strategy*

High frequency events were extracted from ripple, fast ripple and HFO band-filtered data by applying the following algorithmic strategy based on previously reported approaches ^1; 2^. The data comprised multivariate signals consisting of *N* channels. Thus, let ζ*_n_*(t) denote the band-filtered signal of electrode #*n* for *n* = 1,…,*N*. The detection of HFEoI was performed based on the following steps:

1. A mask *m_n_* for thresholding was constructed for each band-pass filtered signal. Let Ω be a compact real interval and *L*^2^(Ω) be the space of square-integrable functions on Ω with |Ω| denoting the Lebesgue measure^3^ of Ω. For *u* in *L^2^*(Ω) let μ[*u*] = 1/|Ω| ∫_Ω_ *u* *dt* denote the mean value of *u* over Ω. Similarly, we define σ[*u*] = |Ω|-1/2 ||*u* - μ[*u*]|| to be the standard deviation of *u* where ||⋅|| denotes the *L^2^*(Ω)-norm. The masking threshold was set at the mean of the signal’s absolute value plus one standard deviation, i.e., *m_n_*(*t*) = ζ*_n_*(*t*), if |ζ*_n_*(*t*)| > μ[|ζ*_n_*|] + σ[|ζ*_n_*|] and *m_n_*(*t*) = 0 otherwise. Since the processing was performed on a channel-by-channel basis, the considerations presented here are discussed in a one-dimensional setting. Hence, for simplicity, the subscript *n* is dropped from now on.
2. A coarse approximate envelope of each signal was constructed based on the smoothed Hilbert transform of the previously computed mask. Thus, with H[*m*] denoting the Hilbert transform of the mask *m*, we computed *m_S_* as the Weierstrass transform^4^ of the absolute value of the analytic signal *m_a_* of *m* given by *m_a_* = *m* + ιH[*m*], where ι denotes the imaginary unit. That is, the envelope |*m_a_*| of *m* was smoothed by convolving it with the Gaussian kernel *G*(*t*) = (4π)^-1/2^ exp(-*t*^2^/4), such that *m_S_* = *g* * |*m_a_*|.
3. A smooth thresholding curve was computed by using the mean value of the approximate envelope over zero-regions of the mask. Hence, we define Ω_0_ ⊂ Ω as the set of points where the mask *m* is zero, which means Ω_0_ = Ω\supp(*m*), where supp(*m*) denotes the support of *m*. Then the mean value of *m_S_* over Ω_0_ is given by *m*_0_ = 1/|Ω_0_| ∫_Ω0_ *m_S_* *dt*. A smooth threshold *θ* for the signal ζ was constructed using *m*_0_ as cutoff value:  *θ*(*t*) = *m_S_*(t), if *m_S_*(t) > *m*_0_, and *θ*(*t*) = 0, otherwise.
4. Signal segments corresponding to non-zero regions of the calculated smooth threshold were classified as HFEoI while the rest of the signal was discarded. Thus, the processed signal ξ was computed as ξ(*t*) = ζ(*t*) for *t* in supp(*θ*) and ξ(*t*) = 0 otherwise. To decrease false-positive count, HFEoI with duration longer than 100 ms were also removed from ξ.

Due to its low-frequency nature, gamma band-filtered data were not subjected to the above detailed HFEoI detection scheme, i.e., ξ(*t*) = ζ(*t*) for all *t* in Ω in the gamma band.

The validity of the presented approach for extracting HFEoIs was verified by comparing modular decompositions of per-second NMI networks obtained using a strategy proposed by Burnos et al.^1^ to community structures calculated based on the algorithm shown above. The Burnos detector is based on two stages: first possible events of interests are detected (high sensitivity, low specificity), in the second stage these events are reviewed to recognize HFOs (low sensitivity, high specificity). The above detailed algorithmic approach was specifically designed to extract HFEoI to then use concepts from information theory to quantify the amount of shared high-frequency information across channels. Thus, the presented HFEoI extraction scheme was compared to the first stage of the Burnos detector whose baseline parameters were adjusted to match the pre-processing setup used here. Instead of five-minute periods of infinite response filtered broad band high frequency data (80-500Hz), Burnos HFEoI detection was applied to the ten-minute segments of finite impulse response HFO-band pass filtered resting state and seizure periods used in the main text. The sensitivity of the Burnos HFEoI detection was further increased by lowering the detection thresholds in Steps 2 and 5 of Stage 1: the signal envelope cutoff was reduced to half the mean envelope plus half its standard deviation, the peak threshold was lowered to half the mean baseline signal plus ¾ of its standard deviation.

*Network Construction*

The NMI between each pair of pre-processed per-second electrode time-courses was calculated as follows. Given a pre-processed recording of duration *T* (in seconds) with sampling frequency *s* (in Hz) let *h* = 1/*s* denote a step-size and let Ω*_h_* = {*t_k_* = *kh* | *k* = 0,…,*sT*} be an equidistant grid on Ω = [0,*T*]. Then Ξ*_i_^K^* = {ξ*_i_*(*t_k_*) | *k* = (*K* - 1)*s* + 1,…,*Ks*} is the time-series of channel *i* (*i* = 1,…,*N*) comprising second *K* (*K* = 1,…,*T*). The *NMI* between Ξ*_i_^K^* and Ξ*_j_^K^* (*i,j* = 1,…,*N*) was calculated by dividing the classic mutual information ^5^ *MI* between Ξ*_i_^K^* and Ξ*_j_^K^* by the geometric mean of the corresponding Shannon entropies ^6^ *H*(Ξ*_i_^K^*) and *H*(Ξ*_j_^K^*) respectively, that is *NMI*(Ξ*_i_^K^*,Ξ*_j_^K^*) = *MI*(Ξ*_i_^K^*,Ξ*_j_^K^*)(*H*(Ξ*_i_^K^*)*H*(Ξ*_j_^K^*))^-1/2^, where we set *NMI*(Ξ*_i_^K^*,Ξ*_j_^K^*) = 0 if Ξ*_i_^K^* = Ξ*_j_^K^* = {0}. All computed pair-wise NMI values *NMI_i,j_^K^* = *NMI*(Ξ*_i_^K^*,Ξ*_j_^K^*) were collected in matrices ***N****^K^*. Hence, for every second *K* a NMI matrix ***N****^K^* was computed for all per-channel time-series Ξ*_i_^K^* (*i = 1,…,N*). Weighted undirected networks were subsequently constructed by directly interpreting each NMI matrix as the connectivity matrix of a weighted undirect graph. In contrast, negative entries in a standard Pearson correlation matrix require either a re-definition of classic graph measures, which may be problematic for path-based metrics due to the possible existence of negative cycles ^7^, or a separate analysis of negative and positive correlation coefficients, which may yield unconnected sub-graphs.

**Results**

*Evolution of Network Topology over Time*

Findings reported in the main text were consistent across all analyzed resting and seizure periods in subjects 1-4. Representative rest and seizure periods of Subjects 2-4 are depicted in Fig. S2-S4. For visualizations of all 48 analyzed recordings (24 seizure and 24 resting states) visit our website at [research.mssm.edu/simonyanlab](../research.mssm.edu/simonyanlab). The topological structure of resting state networks was similar between gamma and high-frequency networks. However, during seizures gamma networks showed changes in community structure only during the ictal period, while high-frequency networks were characterized by pronounced topological variations throughout the entire seizure period, particularly before the electrographic ictal onset (Fig. S2-S4).

*Figure S2 – S4 about here*

Similarly, inter-community migration patterns in HFO networks across all subjects were consistently characterized by nodes rapidly switching modules throughout the time-course of a seizure, while resting state cliques largely retained their topological structure (Fig. S5).

*Figures S5 about here*

*Robustness of High-Frequency Network Architecture with Respect to HFEoI Detection*

The influence of the used HFEoI detection strategy on the modular structure of high-frequency (≥ 80Hz) networks was assessed by generating community maps based on data subjected to the Burnos detector ^1^ instead of the presented HFEoI extraction algorithm. Burnos community maps (Fig. S6) closely resembled results obtained from the presented custom HFEoI detector (compare to Fig.2, S2, S3, S4).

*Figure S6 about here*

**References**

1. Burnos S, Hilfiker P, Surucu O, et al. Human intracranial high frequency oscillations (HFOs) detected by automatic time-frequency analysis. *PLoS One* 2014;9:e94381.

2. Graef A, Flamm C, Pirker S, et al. Automatic ictal HFO detection for determination of initial seizure spread. *Conf Proc IEEE Eng Med Biol Soc* 2013;2013:2096-2099.

3. Ash RB. Real Analysis and Probability. Academic Press: New York; 1972.

4. Hille E, Phillips RS. Functional analysis and semi-groups. American Mathematical Society; 1957.

5. Cover TM, Thomas JA. Elements of information theory. Wiley: New York; 1991.

6. Shannon CE. A Mathematical Theory of Communication. *Bell System Technical Journal* 1948;27:379-423.

7. Fakcharoenphol J, Rao S. Planar graphs, negative weight edges, shortest paths, and near linear time. *Journal of Computer and System Sciences* 2006;72:868-889.

**Supporting Information Legends**

**Figure S1. 3D reconstruction of intracranial (subdural) grid and strip electrode placement in all subjects.** Based on scalp EEG recordings, neuroimaging data and clinical characteristics, subdrual grids were placed on the right fronto-temporo-parietal region. Shown are three-dimensional reconstructions of each subject’s MRI recordings (in native space) with red electrodes illustrating the electrographically determined seizure onset zones.

**Figure S2.** **Functional cortical network structure at different frequency bands during rest and seizure periods for Subject 2.** The spatiotemporal evolution of the modular structure of cortical functional networks in gamma, ripple, fast-ripple and HFO frequency bands during 600 s long rest (A) and seizure periods (B). Each node is assigned a number in the left vertical axis, and each community of nodes (modules) is represented by a color. Starting with electrode #1 at the top, all nodes in the network are shown as a column of pixels. The dimension of each network is *N* = 64, hence every column is 64 pixels long with each pixel shaded using a 6-bit (2^6^ = 64) color palette, representing the maximal possible number of modules. In this manner, all networks are represented by 6-bit pixel columns, which are stacked against each other, starting from the left and advancing in one-second steps in the time dimension to 600. Red vertical lines mark the electrographic seizure onset; yellow vertical lines highlight the end of the ictal period. The overlaid white curves demonstrate the stable temporal evolution of the number of network modules or module size (right axes) at rest in all frequency bands but their significant deviations from resting state values in the seizure period in all high frequency bands. Similar patterns were observed in all other subjects during resting and seizure periods

**Figure S3. Functional cortical network structure at different frequency bands during rest and seizure periods for Subject 3.** Figure caption same as Figure S2.

**Figure S4. Functional cortical network structure at different frequency bands during rest and seizure periods for Subject 4.** Figure caption same as Figure S2.

**Figure S5. Modular affiliation dynamics in functional HFO networks at rest and during seizure periods.** Each panel (A-C) corresponds to resting and seizure periods in Subjects 2-4 also shown in and Video S2, S3, S4. The horizontal axis represents the total number of network modules emerging during rest and seizure periods in each subject with the length of each line segment indicating the corresponding module size. Color-coded arcs mark the peculiar modular affiliation dynamics of each node with the line color indicating the target module. At rest, few dominant modules comprised the network; however, over the time-course of a seizure, numerous small modules fragmented the functional HFO networks (i.e., modular breakdown).

**Figure S6. Community maps based on Burnos HFEoI detection.** Each row of panels shows a community map of functional HFO (100-500 Hz) networks corresponding to a rest (left column) and seizure period (right column) for each subject. Each column illustrates the community affiliation of the current per-second network and every row represents a single network node (#1-#64, left axis) with nodes within a module sharing the same color. The time evolution of the number of detected network modules is illustrated by an overlaid white curve (right axis). Red and yellow vertical lines in the right column mark the electrographic ictal onset and the end of the ictal phase respectively. The dynamic networks were constructed based on the Burnos HFEoI detection scheme and closely resemble results shown in the main text (Fig. 2 for Subject 1) and in the Supporting Information (Figs. S1-S3 for Subjects 2-4).

**Video S1. Spatiotemporal evolution of functional HFO (100-500 Hz) networks during a representative resting (left) and seizure period (right) in Subject 1.** Edge and node color represent NMI coefficient and strength values respectively (normalized between 0 and 1). The video is based on the same resting and seizure periods shown in Figures 2 and 5.

**Video S2. Spatiotemporal evolution of functional HFO (100-500 Hz) networks during a representative resting (left) and seizure period (right) in Subject 2.** Edge and node color represent NMI coefficient and strength values respectively (normalized between 0 and 1). The video is based on the same resting and seizure periods shown in Figure S2.

**Video S3. Spatiotemporal evolution of functional HFO (100-500 Hz) networks during a representative resting (left) and seizure period (right) in Subject 3.** Edge and node color represent NMI coefficient and strength values respectively (normalized between 0 and 1). The video is based on the same resting and seizure periods shown in Figure S3.

**Video S4. Spatiotemporal evolution of functional HFO (100-500 Hz) networks during a representative resting (left) and seizure period (right) in Subject 4.** Edge and node color represent NMI coefficient and strength values respectively (normalized between 0 and 1). The video is based on the same resting and seizure periods shown in Figure S4.

**Table S1. Clinico-pathological characteristics of patients with drug-resistant epilepsy.**

| Subject number | Age/gender | Seizure type | Neuroimaging/Pathological diagnosis |
| --- | --- | --- | --- |
| Subject 1 | 20/M | CPS/SGTC | Mesial Temporal Sclerosis |
| Subject 2 | 25/M | SPS/CPS/Tonic/SGTC | Cortical Dysplasia |
| Subject 3 | 45/M | CPS/SGTC | Cortical Dysplasia |
| Subject 4 | 30/M | CPS/Tonic | Cortical dysplasia |

M: male; CPS: complex-partial seizure, SGTC: secondarily generalized tonic-clonic, SPS: simple-partial seizure.
